# Reasoning and causal inference regarding surgical options for patients with low‐grade gliomas using machine learning: A SEER‐based study

**DOI:** 10.1002/cam4.6666

**Published:** 2023-11-06

**Authors:** Enzhao Zhu, Weizhong Shi, Zhihao Chen, Jiayi Wang, Pu Ai, Xiao Wang, Min Zhu, Ziqin Xu, Lingxiao Xu, Xueyi Sun, Jingyu Liu, Xuetong Xu, Dan Shan

**Affiliations:** ^1^ School of Medicine Tongji University Shanghai China; ^2^ Shanghai Hospital Development Center Shanghai China; ^3^ School of Business East China University of Science and Technology Shanghai China; ^4^ Department of Computer Science and Technology, School of Electronics and Information Engineering Tongji University Shanghai China; ^5^ Department of Industrial Engineering and Operations Research Columbia University New York New York USA; ^6^ School of Ocean and Earth Science Tongji University Shanghai China; ^7^ College of Civil Engineering Tongji University Shanghai China; ^8^ Regenerative Medicine Institute, School of Medicine National University of Ireland Galway Ireland

**Keywords:** causal inference, deep learning, gross‐total resection, low‐grade gliomas, sub‐total resection

## Abstract

**Background:**

Due to the heterogeneity of low‐grade gliomas (LGGs), the lack of randomized control trials, and strong clinical evidence, the effect of the extent of resection (EOR) is currently controversial.

**Aim:**

To determine the best choice between subtotal resection (STR) and gross‐total resection (GTR) for individual patients and to identify features that are potentially relevant to treatment heterogeneity.

**Methods:**

Patients were enrolled from the SEER database. We used a novel DL approach to make treatment recommendations for patients with LGG. We also made causal inference of the average treatment effect (ATE) of GTR compared with STR.

**Results:**

The patients were divided into the Consis. and In‐consis. groups based on whether their actual treatment and model recommendations were consistent. Better brain cancer‐specific survival (BCSS) outcomes in the Consis. group was observed. Overall, we also identified two subgroups that showed strong heterogeneity in response to GTR. By interpreting the models, we identified numerous variables that may be related to treatment heterogeneity.

**Conclusions:**

This is the first study to infer the individual treatment effect, make treatment recommendation, and guide surgical options through deep learning approach in LGG research. Through causal inference, we found that heterogeneous responses to STR and GTR exist in patients with LGG. Visualization of the model yielded several factors that contribute to treatment heterogeneity, which are worthy of further discussion.

## 1 INTRODUCTION

Gliomas are a group of central nervous system (CNS) neoplasms consisting of neuroglial cells, which are the most prevalent malignant brain tumors in adults, with an average annual age‐adjusted incidence of 6 per 1000 people.^1^ Diffuse low‐grade gliomas (LGG), including World Health Organization (WHO) Grade 2 astrocytomas, oligodendrogliomas, and oligoastrocytomas,^2^ account for 15% of all primary brain tumors.^3^

Although LGGs are not considered surgically curable,^4^ the initial treatment usually advocated for LGGs is still maximal safe resection to achieve increased quality of life, prolonged progression‐free survival (PFS), and overall survival (OS).^5^ The extent of resection (EOR) is crucial^6^ and deemed to have a strong correlation with survival time.^7^, ^8^ However, the effect of EOR remains a subject of contention,^8^, ^9^ because of the heterogeneous response to surgery observed in LGG patients^10^, ^11^ based on such as their demographical factors, molecular subtypes,^12^ and tumor location^13^; the absence of randomized control trials (RCT); and the limited availability of robust clinical evidence.^9^, ^14^

An increasing body of literature supports the claim that radical resection significantly improves survival.^15^ Lo et al.^16^ suggested that gross‐total resection (GTR) should be the objective when technically achievable, without causing substantial morbidity. McGirt et al.^11^ found that GTR is independently associated with improved PFS and OS compared to sub‐total resection (STR), with a 25% higher 5‐year survival rate. Other studies^11^, ^17^ have also supported this argument. Nonetheless, there are studies that take a different standpoint. Jung et al.^18^ suggested that when the tumor is located adjacent to or within an eloquent area, GTR is not favored over STR, as it accelerates the malignant transformation of tumors. Other studies have argued that more conservative treatment is recommended, given the inherently long survival time of patients with LGG.^19^, ^20^

Findings from such non‐RCT studies, however, are limited by selection bias, as patients with GTR typically have other good prognostic factors, such as the presence of small, non‐eloquent tumors in otherwise healthy patients.^9^ Estimating unbiased treatment effects in observational studies is challenging because we can only observe correlations, rather than true causality.^21^ An effective but expensive method is to randomly assign interventions (e.g., RCT).^22^ There are also many alternative options for inferring the average treatment effect (ATE), such as two‐stage machine learning (ML) models,^23^, ^24^, ^25^, ^26^ propensity score‐based methods,^27^, ^28^, ^29^ and instrumental variable‐based methods.^30^, ^31^, ^32^, ^33^ To go a step further, the more informative question we wish to clarify is not which is the best choice between GTR and STR, but whether a specific patient would have a better survival outcome with STR or GTR, as clinical treatment is influenced by many underlying features^18^, ^34^, ^35^ and the response of patients to surgery is heterogeneous. The individual treatment effect (ITE) can only be obtained through counterfactual reasoning.^36^ A naive approach is to include treatment as a covariate and artificially change it to obtain ITE; however, the performance of this method can be seriously disturbed by confounders.^37^ Therefore, an individual‐level causal inference^38^, ^39^, ^40^ needs to be conducted.

The objective of this study was to determine the optimal choice between STR and GTR for an individual patient, that is, to improve OS, and to examine features that are potentially relevant to treatment heterogeneity through interpretation of the model to guide clinical intuition of optimal treatment selection with a novel deep learning (DL) approach.

## 2 METHODS

### 2.1 Study design

We conducted a retrospective cohort study to analyze the relationship between surgical treatment and survival outcomes in patients with LGG. The participants included in this study were all selected from the Surveillance, Epidemiology, and End Results (SEER) database, which includes patients with cancer from 18 regions of the United States and represents over 27.0% of the national population.^41^ The Strengthening the Reporting of Observational Studies in Epidemiology (STROBE)^42^ reporting guidelines were followed in this study.

The included patients were diagnosed within the period of 2010–2015. Patients who remained alive on December 31, 2019, were censored. Thus, the follow‐up time ranged from 5 to 10 years. Income is defined as average household income based on zip code.

**FIGURE 1:**
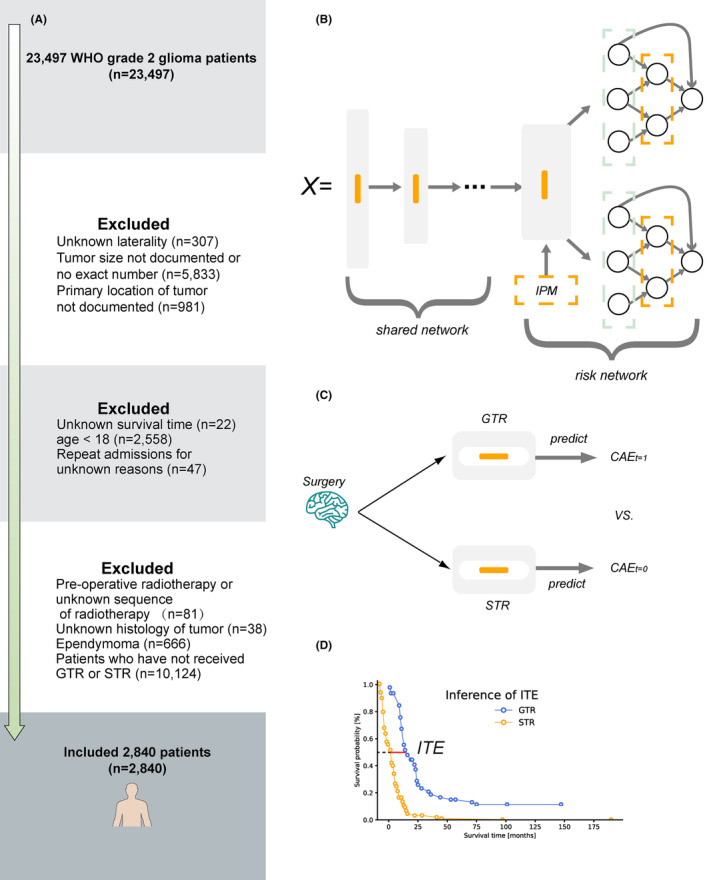
The flow chart of patients' enrollment and the schematic diagram of the balanced survival lasso‐network, T‐learner, and the inference of individual treatment effect. (A) flow chart of patient enrollment; (B) schematic diagram of balanced survival lasso‐network (BSL); (C) schematic diagram of T‐learner; (D) schematic diagram of inference of individual treatment effect (ITE). IPM, integral probability metric; GTR, gross‐total resection; STR, subtotal resection; CATE, conditional average treatment effect; X, features of patients.

Patients were included if they were diagnosed with WHO Grade 2 gliomas treated with STR (SEER code 21: Subtotal resection of tumor, lesion or mass in brain) or GTR (SEER code 30: Radical, total, gross resection of tumor, lesion or mass in brain), defined by MR image, and whose tumor histology is astrocytoma, oligodendroglioma, or oligoastrocytoma. The exclusion criteria were as follows: (1) Ambiguous or unknown tumor laterality and size. (2) Repeat admissions, defined as duplicate Patient ID. (3) Age < 18. (4) Unknown brain cancer‐specific survival (BCSS) time or surgery. The outcome we are interested in is the 5‐ and 10‐year BCSS, which was provided by SEER, indicating the time interval between the diagnosis and final death caused by brain cancer. The predictor variables collected included basic demographic information, including sex, age, marital status, living area, economic status, and reporting state; and clinical pathology information, including tumor location, laterality, extensions, and histology. Figure 1A demonstrates the overall patient inclusion process.

In our study, we used a dataset spanning from 2010 onward. During a portion of this period (2010–2016), the term “Oligoastrocytoma” was still recognized as a distinct entity by the WHO. The removal of this classification occurred in 2016, after which it was recommended to classify these tumors as either astrocytoma or oligodendroglioma based on their molecular characteristics. However, the molecular data necessary for this reclassification is not available for the cases diagnosed prior to this change. As a result, to maintain the integrity of the historical data, we have chosen to retain the term “Oligoastrocytoma” for cases diagnosed between 2010 and 2016.

### 2.2 Machine learning algorithm

For optimal treatment reasoning, we trained two types of models: the two‐learner (T‐learner)^43^ and balanced individual treatment effect (BITES)^38^ framework, which is a recently proposed DL approach. In short, the T‐learner trains an ML model in each of the two treatment populations. Each model represents a hypothesis of treatment during reasoning and yields the conditional average treatment effect (CATE). Unlike T‐learners, BITES uses a shared network, which is a multilayer perceptron (MLP), to extract latent features and uses two risk networks (two MLPs) to represent different treatment assumptions, while these three MLPs are trained end‐to‐end.

In this study, we adapted the architecture of BITES using two LassoNet^44^ to replace risk networks, which we call the **b**alanced **s**urvival **l**asso‐network (BSL). LassoNet is a recent DL method that can be used to handle feature sparsity; it has a linear and a nonlinear part, where linear regression with penalty and complexity control techniques are used to preprocess features, and the nonlinear DL component is then used to process latent features. A feature participates in the DL part only if the weight of its linear counterpart is not zero; thus, the impact of irrelevant features is greatly reduced. We used the feature selection capability of LassoNet to filter the large number of latent features extracted by the shared network to prevent overfitting. The overall model architecture is presented in Figure 1B, and a schematic of the T‐learner is presented in Figure 1C.

For the T‐learner, we trained two separate five‐layered DeepSurv^39^ models on patients who received STR and GTR. We also trained dropouts that met multiple additive regression trees (DART),^45^ regularized coxnet (Rcoxnet),^46^ cox proportional hazards model (CPH), and random survival forest (RSF)^47^ for comparison. All models, except BITES and BSL, were trained and used in terms of T‐learners.

### 2.3 Causal inference on average and individual treatment effect

To estimate the ATE of GTR compared to STR, we used doubly robust learning (DRL)^47^ to derive a two‐stage adjusted logistic regression (LR); the odds ratios (OR) obtained was called OR^d^. First, an LR was used to fit the treatment and response, and another LR was used to predict the response residuals from the treatment residuals, thus making treatment as independent of other covariates as possible. Additionally, we used inverse probability treatment weighting (IPTW)^21^ to reduce treatment selection bias and then obtained the adjusted hazard ratios (HR^a^) of treatment. In IPTW, a prior LR was derived to predict covariates and treatment, and then the propensity score yielded from LR was used to reweigh different treatment groups.

For the ITE estimation, there are two possible treatments, STR and GTR, while only a single factual can be observed and the alternative situation is missing. The T‐learner first estimates the CATE (only for a certain treatment group) and then compares them counterfactually to obtain the ITE of a certain patient. BITES framework models (in this study, BITES and BSL) attempt to learn a balanced representation directly from data through an integral probability metric (IPM),^48^ and use risk networks to mimic T‐learners.

Let the ITE of individual i be defined as ${\mathrm{ITE}}_{i}=Y_{i}\left(X_{i}\;|\;T=1\right)-Y_{i}\left(X_{i}\;|\;T=0\right)$, where T=1 indicates GTR and T=0 indicates STR, and Y is the outcome, which is defined as the length of time that an individual patient's mortality reaches 50% from the beginning. The optimal treatment (model recommendation) was determined based on the ITE value (Figure 1D).

### 2.4 Model development, evaluation, and interpretation

We allocated 75% of the overall patients as the training set for model development and the remaining 25% as the testing set, unseen from the models during the training process, for performance evaluation. For training, we utilized threefold cross validation that trains on two‐thirds of the training set and validates the remaining training set.

Evaluation of models was performed on the testing set. We calculated the concordance index (C‐index) and integrated Brier score (IBS) as regular performance metrics and divided the patients into consistent (Consis.) and inconsistent (In‐consis.) groups based on whether the actual treatment they received was consistent with the model recommendations. Furthermore, we calculated the difference in restricted mean survival time (DRMST) and hazard ratio (HR) as a direct evaluation of the optimal treatment choice recommendation effect.

SHapley Adaptive exPlanations (SHAP)^49^ is a broadly used local interpretation of game theory that explains the extent to which each variable affects the model output with respect to the baseline average. In this study, we conducted SurvSHAP(t),^50^ a time‐dependent SHAP analysis proposed recently, to explain the output of the best model (the greatest recommendation effect) while explaining the differences between the two T‐learners (conditional output head). In addition, we used a linear SHAP analysis to explain the LR that was used to predict recommendations from covariates to guide our intuition of optimal treatment selection.

We also developed a user‐friendly treatment recommendation interface, in which users can input a comma‐separated values (CSV) file that contains features and obtain model recommendations by clicking the “recommend” button. The interface then returns the individual survival probability curve of the counterfactual treatment situation and displays the ITE information.

### 2.5 Statistical analysis

We used R 4.1.3 and Python 3.8 to conduct statistical analysis. Continuous variables were presented as the median and interquartile range (IQR), whereas categorical variables were reported as numbers and percentages (%). LR was used to obtain the OR, and CPH was used to obtain the HR. The log‐rank test was used to compare Kaplan–Meier (KM) curves.

## 3 RESULTS

### 3.1 Baseline information on demographics and clinicopathology

A total of 2840 patients diagnosed with LGG were included in the study, with a median follow‐up time of 38 months (IQR: 15–71) and 24.6% (95% CI: 23.0%–26.2%) occurred BCSS outcome. The median age was 42 years (IQR: 31–55) and 1623 [57.1%] patients were male.

Table S1 shows the baseline demographic and clinicopathological characteristics of patients who received STR and GTR separately. For surgery information, 1245 [43.9%] patients received STR and 1595 [56.2%] received GTR. The median follow‐up time in the STR group was 37 months (IQR: 15–69) and that in the GTR group was 40 months (IQR: 16–72). The BCSS rate of the STR group was 27.6% (95% CI: 25.1%–30.1%) and that of the GTR group was 22.2% (95% CI: 20.2%–24.3%).

### 3.2 Model performance and treatment recommendation

The detailed model performance in the testing set (703 patients) is presented in Table 1. In both STR and GTR groups, BSL achieved the highest C‐index (STR: 0.77, 95% CI, 0.72–0.81; GTR: 0.83, 95% CI, 0.79–0.86) and the best IBS (STR: 0.18, 95% CI, 0.15–0.20; GTR: 0.16, 95% CI, 0.13–0.23).

**TABLE 1:** Detailed model performance.

Model			Overall		STR		GTR	
	DRMST	HR	C‐index	IBS	C‐index	IBS	C‐index	IBS
BSL	4.75 (1.54–7.95)	0.64 (0.49–0.85)	0.80 (0.78–0.83)	0.17 (0.14–0.20)	0.77 (0.72–0.81)	0.18 (0.15–0.20)	0.83 (0.79–0.86)	0.16 (0.13–0.23)
BSL^a^	3.86 (0.64–7.07)	0.68 (0.52–0.90)	0.78 (0.75–0.81)	0.20 (0.16–0.23)	0.75 (0.69–0.79)	0.19 (0.15–0.23)	0.80 (0.76–0.84)	0.19 (0.15–0.24)
BSL^b^	3.77 (0.46–6.89)	0.72 (0.52–0.98)	0.76 (0.75–0.81)	0.18 (0.16–0.20)	0.75 (0.69–0.80)	0.18 (0.15–0.20)	0.79 (0.75–0.84)	0.18 (0.15–0.24)
BITES	3.76 (0.57–6.96)	0.67 (0.51–0.90)	0.75 (0.71–0.79)	0.18 (0.15–0.20)	0.76 (0.71–0.81)	0.20 (0.16–0.25)	0.79 (0.75–0.84)	0.17 (0.14–0.20)
DeepSurv	3.81 (0.63–6.98)	0.77 (0.59–1.04)	—	—	0.66 (0.62–0.71)	0.31 (0.24–0.37)	0.72 (0.67–0.77)	0.24 (0.18–0.29)
CPH	−5.94 (−9.35 to −2.54)	1.62 (0.62–1.23)	—	—	0.74 (0.69–0.79)	0.19 (0.16–0.21)	0.82 (0.78–0.85)	0.17 (0.13–0.20)
RSF	−4.02 (−7.49 to −0.57)	1.33 (1.01–1.77)	—	—	0.70 (0.64–0.75)	0.22 (0.18–0.25)	0.76 (0.71–0.81)	0.17 (0.14–0.19)
Rcoxnet	2.85 (−0.384 to 6.075)	0.74 (0.56–0.97)	—	—	0.76 (0.71–0.81)	0.16 (0.14–0.19)	0.80 (0.76–0.84)	0.16 (0.13–0.19)
DART	−5.05 (−8.44 to −1.65)	1.57 (1.18–2.05)	—	—	0.76 (0.71–0.81)	0.17 (0.15–0.18)	0.81 (0.77–0.85)	0.16 (0.13–0.19)

Abbreviations: BCSS, brain tumor specific survival; BITE, balanced individual treatment effect; BSL, balanced survival lasso‐network; C‐index, concordance index; CPH, Cox proportional hazards model; DART, dropouts meet multiple additive regression trees; DRMST, difference in restricted mean survival time calculated between the Consis. group and In‐consis. group; GTR, patients who underwent gross‐total resection; HR, hazard ratio for model recommendation; IBS, integrated Brier score; IQR, interquartile range; Rcoxnet, regularized coxnet; RSF, random survival forest; STR, patients who underwent sub‐total resection.

^a^ Excluding pathological features.

^b^ Analyzing only patients with astrocytoma or oligodendroglioma.

We used DRMST and HR to directly reflect the differences in BCSS outcomes between the Consis. and In‐consis. groups and to provide cross‐sectional comparisons between the models. We observed the most significant BCSS outcome differences achieved by the recommendation of BSL with a DRMST of 4.75 (95% CI: 1.54–7.95) and an HR of 0.64 (95% CI: 0.49–0.85), followed by BITES (DRMST: 3.76, 95% CI, 0.57–6.96; HR: 0.67, 95% CI, 0.51–0.90) and DeepSurv (DRMST: 3.81, 95% CI, 0.63–6.98; HR: 0.77, 95% CI, 0.59–1.04). There are also examples of models that do not produce positive recommendation effects such as CPH, RSF, and DART.

As a result of the number of each group, in the testing set, BSL determined that 324 [45.6%] patients have taken inappropriate treatment, while 90 [12.8%] patients were deemed to receive STR. 51 [13.0%] patients who would have received GTR were deemed to receive a more conservative surgery, and 272 [87.5%] patients whose actual treatment was STR were deemed to be candidates for GTR by the model.

To ensure clinical utility, we excluded features (histology) acquired postoperatively (BSL^a^). However, the recommendation effect was merely affected (DRMST: 3.86, 95% CI, 0.64–7.07; HR: 0.68, 95% CI, 0.52–0.90). We also did a subset analysis excluding these “Oligoastrocytoma” cases (1245 patients in the training set; 436 patients in the testing set), in which, patients with astrocytoma or oligodendroglioma were analyzed (BSL^b^). Slight performance degradation was observed (DRMST: 3.77, 95% CI, 0.46–6.89; HR: 0.72, 95% CI, 0.52–0.98), which was expected, given the reduction of both training and testing samples. However, the protective effect of BSL remained statistically significant.

We also present the detailed BCSS outcomes of the Consis. group and In‐consis. group in Table S2. Based on the BSL recommendations, the Consis. group achieved the highest 5‐year RMST which was 50.39 (95% CI: 48.37–52.41); the point estimation of median survival time (MST) was beyond the follow‐up time (inf., 95% CI: 162–inf.). The survival probability at 5 years (SaT) was 79.0% (95% CI: 74.0%–85.0%). While the In‐consis. group had the lowest 5‐year RMST (45.56, 95% CI: 43.08–48.04), MST (117, 95% CI: 83–inf.), and 5‐year SaT (65%, 95% CI: 60.0%–71.0%).

**FIGURE 2:**
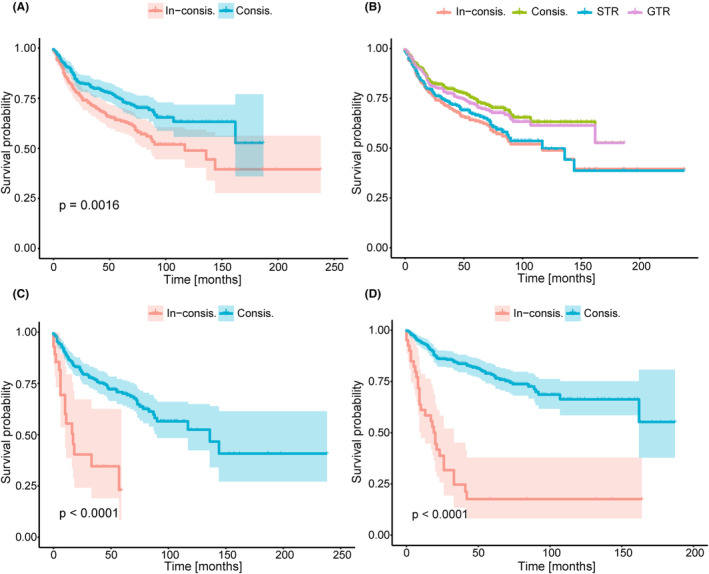
The Kaplan–Meier (KM) curves of different subgroups in the testing set. (A) KM curve of Consis. versus In‐consis. in the testing set; (B) KM curve of Consis., In‐consis., STR group, and GTR group; (C) KM curve of Consis. versus In‐consis. among patients who received STR in the testing set; (D), KM curve of Consis. versus In‐consis. among the patients who received GTR in the testing set. Consis., the actual treatment of the patient was consistent with the model recommendations; In‐consis., the actual treatment of the patient was inconsistent with the model recommendations: GTR, gross‐total resection; STR, subtotal resection.

Additionally, we plotted the KM curve for Consis. versus In‐consis. (Figure 2A); counterfactual recommendation and actual treatment group (Figure 2B); Consis. versus In‐consis. in those whose actual treatment were STR (Figure 2C) and GTR (Figure 2D). Better BCSS outcomes in the Consis. group in every subgroup of patients than that of the In‐consis. group was observed.

### 3.3 Causality of gross‐total resection compared to subtotal resection

**FIGURE 3:**
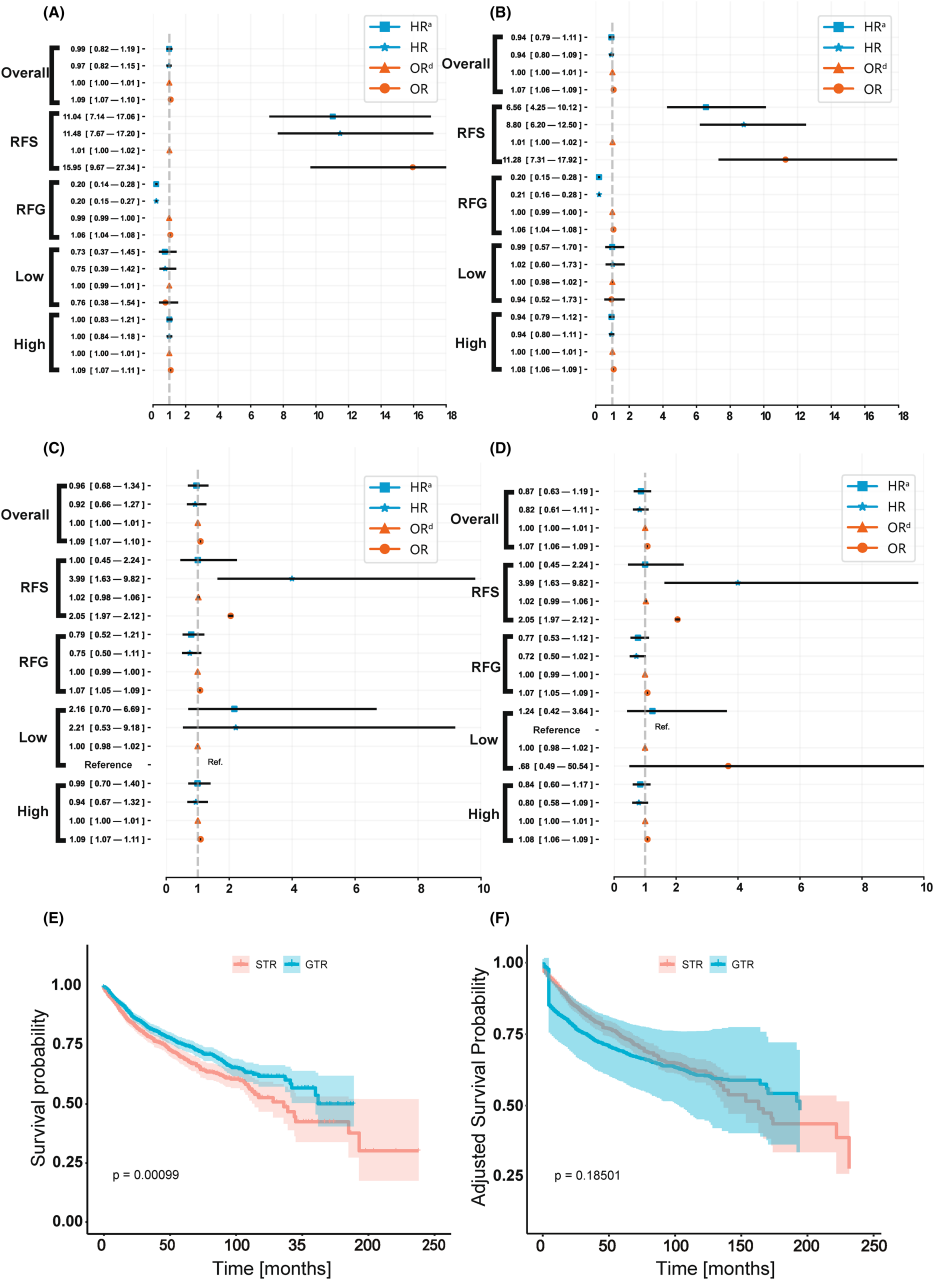
Inference of the average treatment effect of GTR compared to STR in different subgroups. (A) The 5‐year average treatment effect (ATE) in overall patients that included the training and testing sets (OP) of different subgroups; (B) the 10‐year ATE in OP of different subgroups; (C) the 5‐year ATE in the testing set of different subgroups; (D) the 10‐year ATE in the testing set of different subgroups; (E) the Kaplan–Meier (KM) curve of subtotal resection (STR) versus gross‐total resection (GTR) in OP; F, the inverse probability treatment weighting (IPTW) adjusted KM curve of STR versus GTR. Overall, all patients in the OP or testing set; RFS, patients recommended for STR by model; RFG, patients recommended for GTR by model; Low, low‐risk glioma; High, high‐risk glioma. HR, hazard ratio; HR^a^, IPTW‐adjusted HR; OR, odds ratio; OR^d^, doubly robust learning (DRL) adjusted OR.

OR and HR values of GTR compared to STR in 5‐year overall patients that include the training and testing set (OP) and only the testing set are shown in Figure 3A,C, respectively, and the 10‐year values are shown in Figure 3B,D.

Although the point estimate of HR and HR^a^ of GTR was generally smaller than 1, we did not observe a statistically significant result in the OP and testing sets. As for the OR, we found that GTR had higher OR values, but the significance disappeared after correcting for confounders. We also present the unadjusted KM curve (Figure 3E) and IPTW‐adjusted KM curve (Figure 3F) of STR versus GTR in OP. We found that BCSS outcomes in the GTR group were better than STR outcomes before correction (*p* = 0.0010) and that significance was lost after IPTW correction (*p* = 0.1850), which suggests a potential treatment selection bias.

For observations of correlations, we observed high and statistically significant HR and OR values in those who were recommended for STR (RFS) in both the 5‐year and 10‐year OP and testing sets (5‐year HR in testing set: 3.99, 95% CI: 1.63–9.82; 10‐year HR in testing set: 3.99, 95% CI: 1.63–9.82; 5‐year OR in testing set: 2.05, 95% CI: 1.97–2.12; 10‐year OR in testing set: 2.05, 95% CI: 1.97–2.12). Low 5‐year and 10‐year HR values before correction were observed in those who were recommended for GTR (RFG) in OP (5‐year HR in OP: 0.20, 95% CI: 0.15–0.27; 10‐year HR in OP: 0.21, 95% CI: 0.16–0.28) but not in the testing set. We also found a high OR of high‐risk LGG (5‐year OR in OP: 1.09, 95% CI: 1.07–1.11) before DRL correction in both 5‐year and 10‐year values; there were no significant differences found in low‐risk LGG.

As an inference for fact, the significance of both OR^d^ and HR^a^ remained after correction for confounders in 5‐ and 10‐year OP, where GTR was a risk factor for RFS (5‐year HR^a^ in OP: 11.03, 95% CI: 7.14–17.06; 5‐year OR^d^ in OP: 1.01, 95% CI: 1.00–1.02) and as a protective factor in RFG (5‐year HR^a^ in OP: 0.20, 95% CI: 0.15–0.29; 5‐year OR^d^ in OP: 0.99, 95% CI: 0.99–1.00), except for 10‐year OR^d^ in RFS, which was not statistically significant (10‐year OR^d^ in OP: 0.99, 95% CI, 0.99–1.01). Although the point estimates of HR^a^ and OR^d^ in the RFS and RFG groups had approximately the same trend as before correction, the statistical significance was lost. After DRL rectification, the OR^d^ in high‐risk LGG was not statistically significant (5‐year OR^d^ in OP: 1.00, 95% CI: 0.99–1.00; 10‐year OR^d^ in OP: 1.00, 95% CI: 1.00–1.00).

### 3.4 Interpretation of model behavior and recommendation interface

**FIGURE 4:**
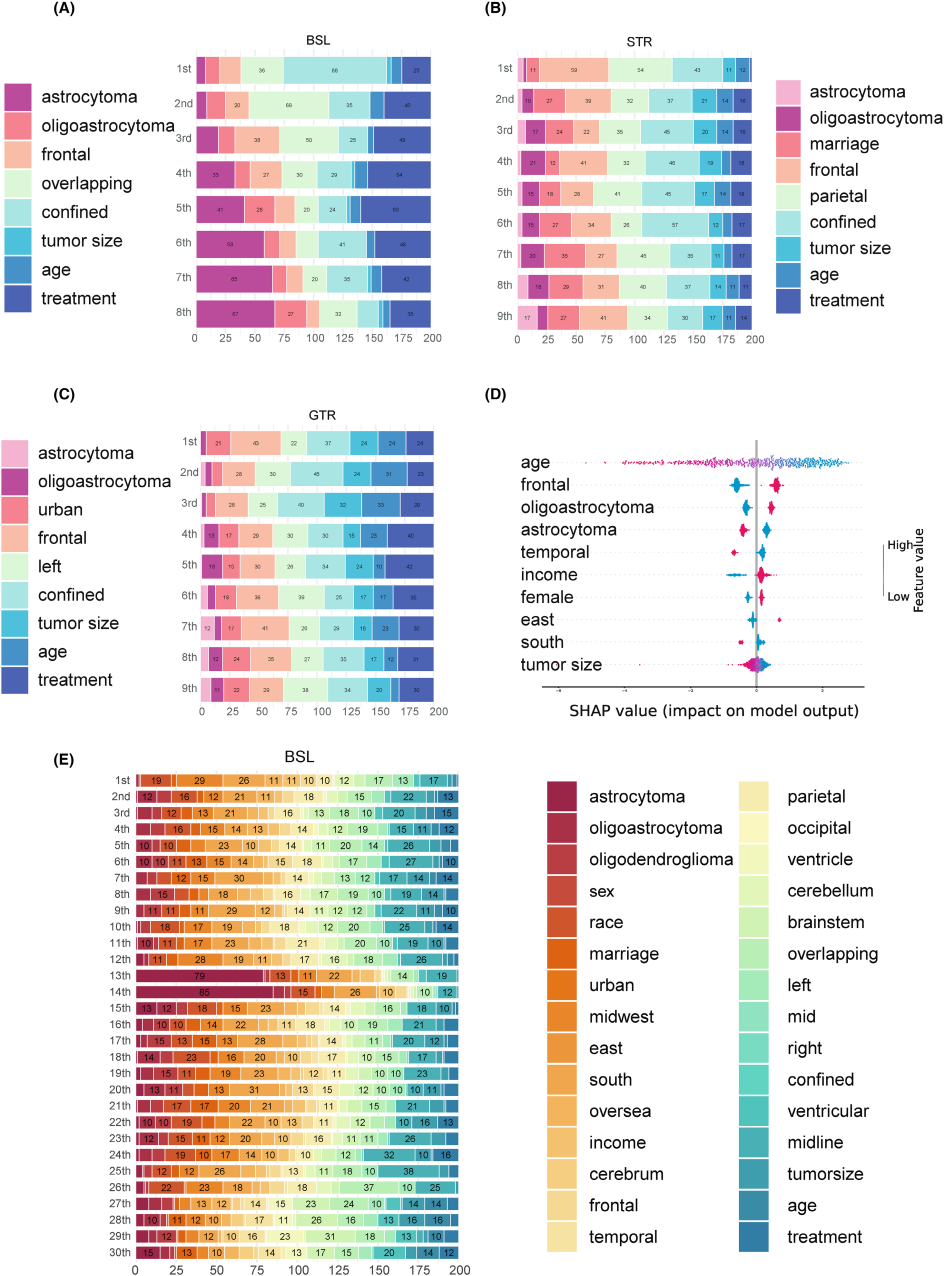
The interpretation of the models using SHapley Adaptive exPlanations (SHAP) analysis. (A) the global rank of the eight most important variables of the balanced survival lasso‐network (BSL); (B) the global rank of the eight most important variables in the subtotal resection (STR) output head of BSL; (C) the global rank of the eight most important variables in the gross‐total resection (GTR) output head of BSL; (D) the recommendation behavior of BSL; (E) the global rank of all variables of BSL.

We used SurvSHAP(t), the first model introduced to date that can explain time‐to‐event DL models to explain the functional output of BSL. The importance of all variables of overall output (the output of two LassoNets were combined) is presented in Figure 4D. For simplicity, we present the eight most important variables, ranked by aggregated SHAP values, of the overall output in Figure 4A. Additionally, we present the eight most important variables of the conditional output head in Figure 4B,C for STR and GTR, respectively. The linear SHAP summary plot of the recommendation behavior of the BSL is shown in Figure 4E.

We sampled 200 observations from the testing set to visualize the BSL. In SurvSHAP(t), horizontal bars represent the number of observations for which the importance of the variable, represented as a given color, was classified as first, second, and so on. In BSL, “treatment” is not entered into the model as a covariate, but as a marker for the output of the observations from the different LassoNets and using different baseline hazards based on treatment groups.

As for the interpretation of the overall output, confined was deemed the most important by 88 samples. Voted by the majority, overlapping is the second most important, followed by frontal tumor location and histology of astrocytoma and oligoastrocytoma. For conditional output, we observed that marriage and parietal location, considered the top eight important variables in the STR output head, were replaced by urban and left laterality. The STR output head paid more attention to frontal, parietal, confined, marriage, and tumor size; except for frontal location and confined, the GTR output head took equal note of age and left laterality.

Linear SHAP showed a more pronounced trend toward treatment recommendations. A red dot indicates a larger value (or presence) of this variable, whereas a larger SHAP value indicates that an increase (or decrease) in this variable drives the model (fitted by an LR) to recommend GTR. We presented the 10 most important variables ranked by aggregated linear SHAP values. Age was deemed the most important; broadly speaking, the greater the age, the more the GTR is not recommended. The presence of frontal, oligoastrocytoma, higher income, female sex, treated in the east of the United States, and left laterality led to the recommendation of the GTR. Conversely, astrocytoma, temporal, being treated in the south of the United States, and large tumor size require more conservative treatment.

**FIGURE 5:**
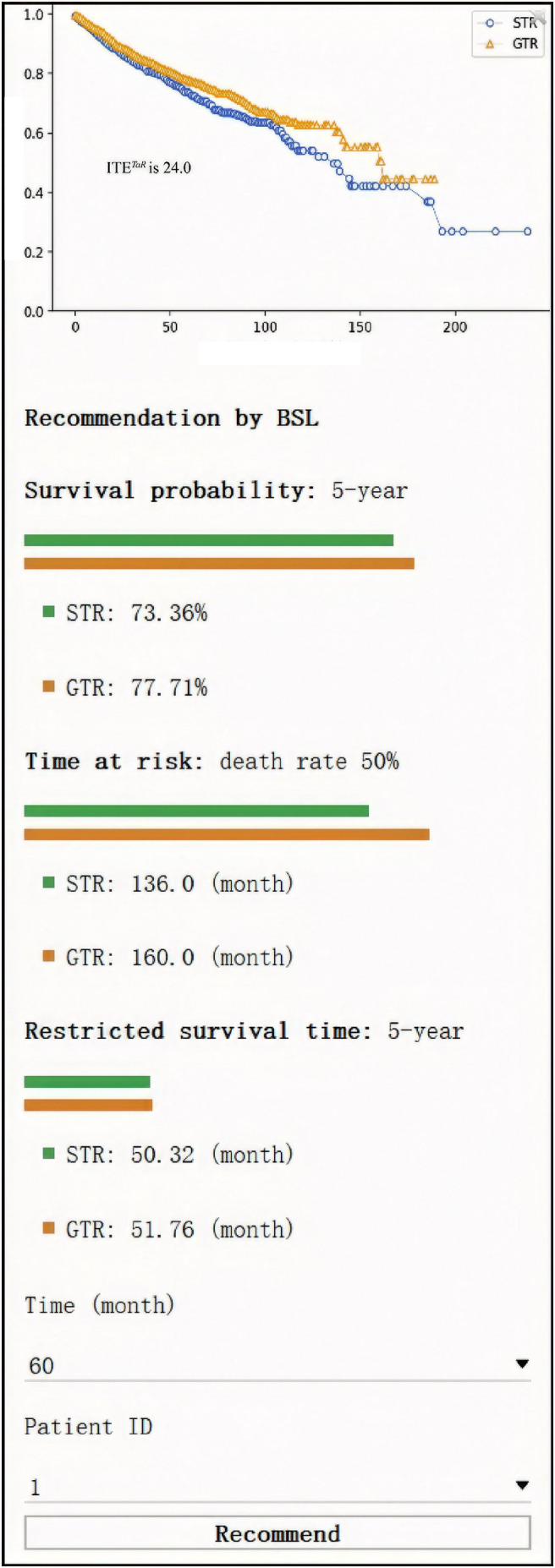
Treatment recommendation interface. STR, subtotal resection; GTR, gross‐total resection; ITE, individual treatment effect; TaR, difference between time to 50% mortality in an individual patient receiving GTR and STR.

Additionally, we demonstrate a user‐friendly surgery recommendation interface in Figure 5, in which the user can upload a CSV file with patient features and obtain the recommendation results easily. In this interface, the ITE and the potential survival outcomes of patients under different treatments will be demonstrated to assist clinicians and patients in weighing the pros and cons and making surgical choices.

## 4 DISCUSSION

The EOR is crucial for WHO Grade 2 glioma (LGG) as an initial treatment^14^; however, the true correlation between EOR and survival outcomes remains a matter of great debate.^8^, ^51^, ^52^ There are many heterogeneous subgroups within the LGG patient population, such as high‐ and low‐risk LGG,^53^ different tumor locations, and different tumor sizes. The responses of these subgroups to surgery can be heterogeneous. For ethical reasons, we were unable to assign STR to a patient with LGG who would otherwise be considered suitable for GTR based on current clinical experience. Therefore, current RCT studies are lacking, and we can only analyze the advantages and disadvantages of STR and GTR through observational studies.^9^, ^54^ Bias exists in that different treatment groups may have different prognostic characteristics; for example, patients suitable for GTR surgery may have healthier survival or pathological features.^9^

Furthermore, when considering which of the two procedures is better for a particular patient, it is impossible to observe in reality and draw conclusions, as each patient is unique and has only received one treatment. An expensive and complex method is to continually subdivide subgroups through clinical experience to approximate an individual patient or a particular class of patients. A novel and effective approach implemented in this study is to use ML to infer ITE and guide clinical experience by interpreting ML models.

BITES^38^ is a recently proposed DL approach that learns balanced generating representations^55^ and uses T‐learners to predict CATE. In this study, we restructured BITES to replace risk networks with two LassoNets,^44^ called **b**alanced **s**urvival **l**asso‐network (BSL). With this simple modification, the BSL achieved the best discrimination. We were more interested in the effectiveness of the treatment recommendations. As a result, BSL performed best, which can be evidenced by the lower HR values (0.64, 95% CI: 0.49–0.85) and higher DRMST (4.74, 95% CI: 1.53–7.94); in other words, patients treated in line with the model's recommendations can reduce the risk of death by 36% and the average survival time within the 5‐year period will be longer by 4.74 months. In the testing set, better survival outcomes were observed in the Consis. group, both in the overall patient population (*p* = 0.0016) and in patients who originally received GTR (*p* < 0.0001) or STR (*p* < 0.0001). We also observed some widely used models that did not make good recommendations, such as CPH. This situation may be related to the linear assumption.^39^ A total of 325 [45.6%] patients in the testing set were deemed to have received improper treatment, which indicated that there are very likely to be potential features that make a patient heterogeneous for treatment that is undetected by clinicians.

We further analyzed the overall ATE and several subgroups of patients with LGGs. The survival outcome of the GTR group was significantly better than that of the STR group (*p* = 0.0010) before IPTW correction, however, after correcting for all other confounders, this advantage could no longer be observed (*p* = 0.1850). This result confirmed the speculation mentioned above that patients who undergo GTR may inherently have a more optimistic survival outcome.^9^ Another possible but not yet researched hypothesis raised by Jiang^9^ is that conservative management may be more suitable for low‐risk patients, and immediate resection may cause higher mortality in high‐risk patients. In this study, we did not observe statistically significant results for low‐risk LGG but found a slightly higher OR in the high‐risk group. Unfortunately, we were unable to confirm the time of surgery. In OP, we found a strong heterogeneous response in RFS and RFG. Even after correction, GTR (compared to STR) showed a strong protective factor for RFG (5‐year HR^a^: 0.20, 95% CI, 0.14–0.27) and became a strong risk factor for RFS (5‐year HR^a^: 11.04, 95% CI, 7.14–17.06), indicating that subgroups with strong heterogeneity were found.

Therefore, we interpreted BSL using SurvSHAP(t) and analyzed the recommendation behavior using linear SHAP. The feature importance of BSL generally matches clinical experience and previous research.^1^, ^56^, ^57^ As mentioned previously, a normal way to assess which characteristics of individual patients or a particular group of patients are suitable for which procedure is through clinical consensus.^58^ With the help of individual‐level causal inference, our results showed a clear tendency for GTR to be recommended for patients with frontal location,^59^ oligoastrocytoma histology, female sex, smaller tumor volume,^59^, ^60^ etc.; STR will otherwise be recommended for patients with astrocytoma, temporal location, older age, etc. Given that consistency with the model's recommendations allowed patients to have longer survival times, the results generated by the interpretation of the model in this study are worthy of validation by subsequent studies.

This study has some limitations. Due to database restrictions, we were unable to access some important features, such as isocitrate dehydrogenase (IDH) gene mutation, 1p/19q codeletion status, and cranial imaging information. Given that IDH mutation and 1p/19q codeletion status are highly important prognostic markers concerning the survival of LGG patients, we strongly advocate conducting further research to delve into this topic, contingent upon the availability of pertinent data regarding IDH mutation and 1p/19q codeletion status. Also, we were unable to apply the 2021 WHO classification of CNS tumors, which can compromise the protective effect of the existing model and reduce its clinical utilization value. In addition, the choice of surgery in LGG patients is highly subjective and requires consideration of many factors such as quality of life, surgeon preference, family support, etc. Therefore, future work based on additional characteristics and extensive assessment of patients' outcomes is needed. Finally, while our findings are significant within the context of the SEER database, further validation using diverse datasets is crucial to ascertain the generalizability of our results.

In conclusion, to the best of our knowledge, this is the first study to utilize the DL approach to counterfactually infer ITE and recommend treatment in LGG patients. Patients who were consistent with the model recommendations had significantly better survival outcomes than those who were not. Through causal inference, we found that heterogeneous responses to STR and GTR exist in patients with LGG. Visualization of the model yielded a number of factors that contributed to treatment heterogeneity, which are worthy of further discussion. As this is the first study to make causal inferences about the heterogeneity in patients with LGG, we have not discussed the conclusions obtained in detail, which will be addressed in future research.

## AUTHOR CONTRIBUTIONS

**Enzhao Zhu:** Conceptualization (lead); formal analysis (lead); methodology (lead); writing – original draft (lead). **Weizhong Shi:** Conceptualization (equal); formal analysis (equal); funding acquisition (equal). **Zhihao Chen:** Conceptualization (equal); formal analysis (equal); methodology (equal); writing – original draft (equal). **Jiayi Wang:** Formal analysis (equal); methodology (equal). **Pu Ai:** Formal analysis (equal); methodology (equal). **Xiao Wang:** Formal analysis (equal); methodology (equal). **Min Zhu:** Formal analysis (equal). **Ziqin Xu:** Data curation (equal). **Lingxiao Xu:** Formal analysis (equal). **Xueyi Sun:** Data curation (equal). **Jingyu Liu:** Formal analysis (equal). **Xuetong Xu:** Formal analysis (equal). **Dan Shan:** Project administration (lead); writing – review and editing (lead).

## CONFLICT OF INTEREST STATEMENT

All authors declare no conflict of interest.

## ETHICS STATEMENT

The studies involving human participants were approved by the national cancer institution. Written informed consent for participation was not required for this study in accordance with the national legislation and the institutional requirements.

## Supporting information


Table S1:
Table S2.

## Data Availability

This study analyzed public datasets which can be found here: the Surveillance, Epidemiology, and End Results Program (https://seer.cancer.gov/index.html).
