# Supplementary material for: Reasoning and causal inference regarding surgical options for patients with low‐grade gliomas using machine learning: A SEER‐based study
Source: Cancer Med. 2023 Nov 6;12(22):20878–91. doi: 10.1002/cam4.6666 (PMC10709720; doi:10.1002/cam4.6666)
Supplement: Supplementary file 1 — Table S1: Table S2. [file CAM4-12-20878-s001.docx]

Table S1: Baseline demographic and clinicopathological information

|  | STR  (n=1,245) | GTR  (n=1,595) |
| --- | --- | --- |
| Age, median (IQR), y | 43 (32–55) | 41 (31–54) |
| Tumor size, median (IQR), mm | 49 (34–62) | 40 (28–55) |
| Sex |  |  |
| Male | 702 (56.4%) | 921 (57.7%) |
| Race |  |  |
| White | 1057 (84.9%) | 1394 (87.4%) |
| Married |  |  |
| Yes | 680 (54.6%) | 885 (55.5%) |
| Urban |  |  |
| Yes | 1,107 (88.9%) | 1,422 (89.2%) |
| Area of U.S. |  |  |
| Midwest | 904 (72.6%) | 1,072 (67.2%) |
| East | 136 (10.9%) | 230 (14.4%) |
| South | 190 (15.3%) | 285 (17.9%) |
| Oversea | 15 (1.2%) | 8 (0.5%) |
| Income |  |  |
| Higher than $55,000 | 990 (79.5%) | 1,241 (77.8%) |
| Histology |  |  |
| Astrocytoma | 525 (42.2%) | 668 (41.9%) |
| Oligoastrocytoma | 491 (39.4%) | 675 (42.3%) |
| Oligodendroglioma | 229 (18.4%) | 252 (15.8%) |
| Location |  |  |
| Frontal | 564 (45.3%) | 880 (55.2%) |
| Temporal | 275 (22.1%) | 308 (19.3%) |
| Parietal | 151 (12.1%) | 185 (11.6%) |
| Occipital | 15 (1.2%) | 26 (1.6%) |
| Cerebellum | 28 (2.2%) | 22 (1.4%) |
| Brainstem | 12 (1.0%) | 7 (0.4%) |
| Ventricle | 15 (1.2%) | 19 (1.2%) |
| Overlapping | 134 (10.8%) | 124 (7.8%) |
| Laterality |  |  |
| Left | 594 (47.7%) | 728 (45.6%) |
| Mid | 85 (6.8%) | 73 (4.6%) |
| Right | 566 (45.5%) | 794 (49.8%) |
| Tumor extension |  |  |
| Confined | 606 (48.7%) | 878 (55.0%) |
| Ventricles | 26 (2.1%) | 20 (1.3%) |
| Midline | 92 (7.4%) | 64 (4.0%) |
| BCSS outcome | 27.6 (25.1%–30.1%) | 22.2% (20.2–24.3%) |

STR, patients who underwent sub-total resection; GTR, patients who underwent gross-total resection.

BCSS, brain tumor specific survival; IQR, interquartile range.

Table S2: Detailed recommendation effect.

| Model | Consis. | | | In-consis. | | | p value |
| --- | --- | --- | --- | --- | --- | --- | --- |
|  | RMST | MST | SaT | RMST | MST | SaT |  |
| BSL | 50.39 (48.37–52.41) | inf. (162–inf.) | 0.79 (0.74–0.85) | 45.56 (43.08–48.04) | 117 (83–inf.) | 0.65 (0.60–0.71) | 0.0016** |
| BSL^a^ | 49.86 (47.82–51.89) | 162 (136–inf.) | 0.74 (0.69–0.79) | 45.99 (43.51–48.49) | 117 (83–inf.) | 0.67 (0.62–0.73) | 0.007** |
| BSL^b^ | 49.46 (46.79–52.13) | inf. (90–inf.) | 0.68 (0.62–0.75) | 44.70 (41.41–47.98) | 88 (72–inf.) | 0.61 (0.53–0.69) | 0.05* |
| BITES | 49.64  (47.56-51.71) | inf. (162–inf.) | 0.77  (0.73-0.82) | 46.10  (43.67-48.53) | 117 (83–inf.) | 0.66  (0.60-0.72) | 0.0058** |
| Deepsurv | 50.00  (47.92-52.08) | 162  (117–inf.) | 0.76  (0.71-0.81) | 46.21  ( 43.82-48.61) | 144  (92–inf.) | 0.67  (0.62-0.73) | 0.088 |
| CPH | 43.34  (40.49-46.24) | inf. (162–inf.) | 0.62  (0.55-0.69) | 50.29  (48.43-52.15) | 107  (78–inf.) | 0.77  (0.73-0.82) | 0.00059** |
| RSF | 45.48  (42.57-48.40) | 162  (136–inf.) | 0.68  (0.62-0.75) | 49.52  (47.64-51.38) | 144  (89–inf.) | 0.73  (0.69-0.78) | 0.046* |
| Rcoxnet | 49.35  (47.31-51.40) | 117 (87–inf.) | 0.76  (0.71-0.81) | 46.51  (44.01-49.01) | inf. (162–inf.) | 0.67  (0.61-0.73) | 0.031* |
| DART | 44.93  (42.11-47.76) | inf. (144–inf.) | 0.65  (0.60-0.73) | 50.00  (48.12-51.87) | 107 (78–inf.) | 0.77  (0.72-0.82) | 0.0016** |

BSL, balanced survival lasso-network; BITE, balanced individual treatment effect; CPH, Cox proportional hazards model; RSF, random survival forest; Rcoxnet, regularized coxnet; DART, dropouts meet multiple additive regression trees. a, excluding pathological features; b, analyzing only patients with astrocytoma and oligodendroglioma.

RMST, restricted mean survival time; MST, median survival time; SaT, survival probability at 5 years after diagnosis; p value, p value of log-rank test; inf., not reached within the observation time.

Consis., patients received the same treatment as recommended; In-consis., patients received the alternative treatment as recommended.

*, p value < 0.05; **, p value < 0.01.
